# Mining the hidden treasures from canid genomes

**DOI:** 10.1093/nsr/nwy087

**Published:** 2018-08-30

**Authors:** Fangqing Zhao

**Affiliations:** Beijing Institutes of Life Science, Chinese Academy of Sciences, China

Mining genetic variation including structural variation from mammalian genomes is a crucial step towards investigating the relationship between genotype and phenotype. However, compared to the detection of single nucleotide variants and small indels, characterizing large and particularly complex structural variation is much more difficult and less intuitive [1]. In the past few years, the application of high-throughput-sequencing technologies has greatly facilitated the decoding of canid genomes and unveiled their genetic repertoire and domestication histories [2–5]. However, the genetic landscape of canid structural variation and how are they involved in dog domestication are still poorly understood.

In the paper, Wang *et al.* presented high-quality draft genomes of the grey wolf (*Canis lupus*) and dhole (*Cuon alpinus*), and then identified a large number of dog-specific structural variants [6]. Functional annotation of the genes associated with these dog-specific structural variants indicates that they are enriched in energy metabolisms, neurological processes and immune systems. Interestingly, the authors found that AKR1B1, encoding an enzyme that catalyses the reduction of glucose to sorbitol using NAD(P) H as a cofactor, was duplicated in the dog genome. They speculated that the copy number gain of AKR1B1 gene during dog domestication may be associated with dogs' adaption from a carnivorous diet to a starch diet. This study provides a rich resource of canid structural variants and shed novel insights into the genetic basis of dog domestication.
